# An Inherent Instability of Efficient Markets

**DOI:** 10.1038/srep02784

**Published:** 2013-09-27

**Authors:** Felix Patzelt, Klaus Pawelzik

**Affiliations:** 1 Institute for Theoretical Physics, University of Bremen, Germany

## Abstract

Speculative markets are often described as “informationally efficient” such that predictable price changes are eliminated by traders exploiting them, leaving only residual unpredictable fluctuations. This classical view of markets operating close to an equilibrium is challenged by extreme price fluctuations which occur far more frequently than can be accounted for by external news. Here we show that speculative markets which absorb self-generated information can exhibit both: evolution towards efficient equilibrium states as well as their subsequent destabilisation. We introduce a minimal agent-based market model where the impacts of trading strategies naturally adapt according to their success. This implements a learning rule for the whole market minimising predictable price changes, and an extreme succeptibility at the point of perfect balance. The model quantitatively reproduces real heavy-tailed log return distributions and volatility clusters. Our results demonstrate that market instabilities can be a consequence of the very mechanisms that lead to market efficiency.

Social systems self-organise. In consequence, collective dynamics can emerge that serve to pursue a common goal which is actually not present in the behaviour of the single agents^1^. Financial markets are examples for such systems where the rules for the agents' interactions and agent objectives (generating profit) are well defined. The view that markets in fact operate in distinguished equilibrium states became influential in economics^2^. Here, a fundamental hypothesis is that prices “fully reflect available information”^3^, or at least come close to this limit^4^, such that risk-free (arbitrage) profits cannot be made by (re-)using said information. That is, information that has already been available to the market no longer gives rise to price changes and therefore cannot be exploited. If true, one of the implications of this “Efficient Market Hypothesis” (EMH) is that resulting prices fluctuate randomly^5^. Empirical findings in favour of the Efficient Market Hypothesis include the general absence of exploitable autocorrelations among price changes in financial markets^6^.

The magnitudes of price changes (“volatilities”), however, are found to be correlated for long periods of time. That is, large price changes are typically followed by large ones and small changes by small ones, indicating that markets are complex dynamical systems involving long memories^7^,^8^,^9^. Furthermore, logarithmic price changes (log returns) exhibit large fluctuations even in the absence of external news^10^,^11^. This contradicts expectations for efficient market equilibria where price changes were hypothesised to always reflect external news^12^. More precisely, log return distributions exhibit heavy tails where events that are many times bigger than the standard deviation occur at a much higher frequency than what would be expected if they were Gaussian distributed^7^,^8^,^13^ (Fig. 1 (c)). These so-called stylised facts of real price time series hint at an underlying system that is considerably self-referential^14^. They were associated with substantial market inefficiencies including herding effects^15^, “bubbles”, or with the interactions of heterogeneous traders with limited rationality^12^,^16^ in a market exactly at a critical point^17^.

Here we investigate whether the apparent antinomy of stabilising information-efficient control and dynamics resembling systems operating close to criticality can be resolved. It was recently demonstrated that adaptive control of a dynamical system can generically lead to an instability where the susceptibility to noise dramatically increases close to the point of perfect balance^18^. This principle would apply to markets if two requirements were fulfilled: First, markets need to absorb information about predictable price changes. Ideally, this property should hold independently of the rationality of the individual traders, which cannot be guaranteed. Second, a self-referential market would have to become susceptible to residual noise once all locally relevant information has been exploited.

The first property is a rather common view in economics. If a profit opportunity is present, investors will increasingly exploit it until their price impacts cancel said opportunity. For example, if a certain stock is priced too low, traders will increase buy orders and thereby raise its price. The second property is also intuitive. As traders try to detect trends or patterns in the price dynamics, they effectively predict how the market will react to available information. However, once the agents' actions have led to a balanced equilibrium, it becomes increasingly difficult to distinguish predictable price fluctuations from random noise. If traders then continue to act upon the random fluctuations as if they would hold meaningful new information, their actions will not be balanced anymore. That is, it may be impossible to predict whether a group of traders will overreact to the supposedly new information and to attenuate the resulting price jump by exploiting it. Therefore, atypically large price movements may become much more likely than expected for a Gaussian distribution.

## Results

As a concrete example of the fundamental dynamical instability arising from information absorption as it may be realised in financial markets, we introduce a minimal agent-based trading model. Each agent is representative of one trading strategy and possesses two types of assets which are called money and stocks in the following. For simplicity, we consider a coarse-grained price time series where one step could be considered as e.g. a day.

In each trading step, each agent either offers an amount of money to buy stocks or an amount of stocks in exchange for money. The decision which action to take depends deterministically on the information state and on the agent's strategy. The fraction of an agent's money that is used to buy stocks (or of stocks offered to receive money, respectively) is denoted by the use parameter *γ*. The absolute amount of the asset that an agent offers therefore also depends on how much of this asset the agent holds at the time the offer is made.

At each time *t* the price *p*(*t*) is determined from the ratio of the totalities of demand and supply, respectively. Finally, all trades are performed at this price *p*(*t*), similar to market orders except that here the exchange of money and stocks is executed synchronously for all orders of all agents at the same time *t* (for the mathematical formulation, see the supplement). Note that this simple trading rule conserves the total amounts of each asset, money and stocks, as it should if trading fees can be neglected.

Agents base their decisions on public information states. In a speculative market, traders react to information that can originate from both past prices and any kind of external news. Both may potentially influence the agents' behaviour and therefore might ultimately lead to price changes which could be exploited. Therefore, we use a consistent representation for both endogenous and exogenous information. In each time step *t* one of *D* possible states, which is denoted by an index *μ*(*t*) ∈ {1, …, *D*}, is conveyed to the agents^17^. Each agent's decision to either buy or sell is predetermined at random for each *μ*. We consider two different methods for the generation of these information states:

For endogenous information, agents possess a memory of the most recent *K* signs of the log returns which indicate whether the prices *p*(*t* − *K*), …, *p*(*t* − 1) decreased or increased with respect to their predecessors. This information can take one out of *D* = 2*^K^* possible states. For example, if agents had a memory of the past *K* = 2 returns they could distinguish between *D* = 4 possible combinations of subsequent price movements: (up, up), (down, down), (up, down), and (down, up). The public information *μ*(*t*) ∈ {1, 2, 3, 4} then encodes which one of these 4 states describes the immediate history of the system. That is, it describes the direction of the price change from time *t* − 3 to *t* − 2, and of the price change from *t* − 2 to *t* − 1. This information is publicly available and can be considered to provide an important information about the state of the market.

For exogenous information we use the same notation but envision a binary encoding of external information. For simplicity, the *μ*(*t*) here are drawn randomly and independently with probability *P*_ext_(*μ*). Unless stated otherwise, all *μ* have equal probabilities *P*_ext_(*μ*) = 1/*D*. We also investigated mixed information and obtained results similar to the endogenous case (see supplement).

We focus on markets that are dominated by speculators who can only win or lose assets by betting on price changes within the market. To investigate the effect of a small number of traders that convey new assets to the market or draw out their profits, we divide the agents into *N_s_* speculators and *N_p_* producers. In contrast to the set of agents representing the speculators, the producers' resources are defined to remain constant. Since the strategies of the producers are chosen in the same way as for the speculators, they also deterministically depend on the information state. Thereby the producers contribute predictable amounts of liquidity and stocks to the market. These demands and supplies can then be exploited by the speculators, who are competitive and redistribute their possessions. Initially, all agents are provided with equal amounts of assets.

**Figure 1:**
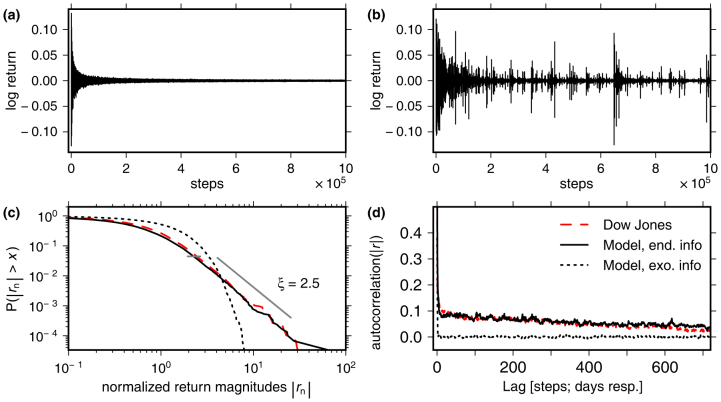
Analysis of log returns *r*(*t* + 1) = log*p*(*t* + 1) − log*p*(*t*), where *p*(*t*) is the price at time step *t*. (a): Time series of the trading model with uniformly distributed exogenous information. Parameters: *N_s_* = 2^10^, *N_p_* = 0, *D* = 2^9^, *γ* = 0.8. (b): Time series for the same model, but with endogenous information. (c): Complementary cumulative distribution function of log return magnitudes obtained by rank ordering. Dotted black: The same simulation as in (a). Solid black: the same simulation as in (b). Dashed red: Daily returns for the Dow Jones Industrial Average (DJIA) since 1900. Short grey line: Hill estimator for the scaling exponent *ξ* of the black curve, where large returns show power-law scaling *P*(|*r*| > *x*) ∝ *x*^−*ξ*^. The cutoff optimises the Kolmogorov-Smirnov statistic^18^. For a fair comparison, 3 · 10^4^ time steps after transients were analysed for the simulations to match the length of the Dow Jones time series. Each time series was normalised by dividing by its standard deviation. (d): Autocorrelations of the log-return magnitudes |*r*|. Line styles are identical to (c). Positive autocorrelations for the model with endogenous information, and the DIJA persist for more than 2 years.

Log-return time series for the model with exogenous information are shown in Figure 1 (a). A strong reduction of initial fluctuations is observed, leaving only a narrow band of Gaussian distributed returns after the transient. Figure 1 (b) shows the endogenous case. Here, in contrast, initial return magnitudes are reduced only in the mean. The magnitudes of the few most extreme returns, however, are less reduced. The remaining fluctuations are analysed in Figure 1 (c), where cumulative distributions of return magnitudes are shown for both cases and compared to the Dow Jones Industrial Average (DJIA). The latter serves as an example for a typical price time series. For the endogenous case, the distribution tail is well described by a power-law and in good agreement with the DJIA. Return fluctuations in the endogenous case also tend to form clusters in time. This effect is quantified by long-range temporal correlations of return magnitudes shown in Figure 1 (d) and is also consistent with the DJIA. More examples for the consistency of the volatility in the model with real data are shown in the supplement.

To understand the model dynamics, we first consider the exogenous case which is fully analytically tractable. The rules of asset redistribution by trading are equivalent to a learning rule related to gradient descent where *γ* is a learning rate. Therefore, the market as a whole minimises predictable price changes. The reason for this stabilising control is that trading success increases the impact of agents whose actions contribute to a reduction of price fluctuations. Note that since agents represent trading strategies and not individual traders, *γ* determines how fast relative impacts of different strategies are adjusted; it does not necessarily reflect how much of their resources actual traders would put at risk.

A phase transition with respect to the critical parameter *α* = *D*/*N_s_* is identified at *α* = 1/2, the point where random binary vectors (the agents) with positive weights (the assets) form a complete basis in the *D*-dimensional strategy space in the limit *N_s_* → ∞. Beyond this point, a speculative market without producers evolves the distribution of assets onto a manifold where the price is invariant to trading. That is, agents still trade and exchange assets, but the price remains constant. Markets that include producers still exhibit finite returns also for *α* < 1/2. Otherwise, for *N_p_* ≪ *N_s_*, return distributions only depend weakly on *N_p_*. See the supplementary material for more details.

These results establish that the market as a whole tends to attenuate its responses (i.e. the magnitudes of the returns) to information states that are presented i.i.d. and in random order. Because this is straightforwardly related to learning via the adaptation of the agents' possessions, we term this effect “information annihilation”: a repeatedly presented information state no longer carries information about exploitable price changes. The relation to Shannon information is discussed further below.

**Figure 2:**
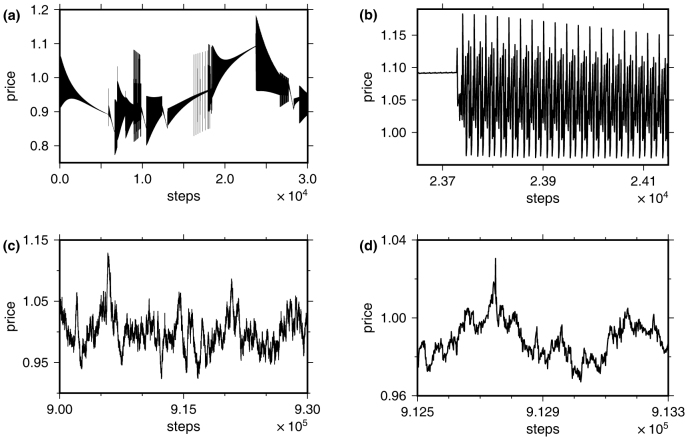
(a): Price time series with a very slow rate of resource redistribution (use) *γ* = 0.01. Other parameters: *N* = 2^10^, *N_p_* = 2^4^, *D* = 2^9^. (b): A zoom in on the time series shown in (a). (c): Price time series with *γ* = 0.8, other parameters equivalent to (a). (d): A zoom in on the time series shown in (c).

When the *μ*(*t*) are endogenously generated, the same mechanism of information absorption present in the exogenous case ensures that the system relaxes towards local price equilibria and returns vanish, but only transiently. To illustrate this basic principle, Figure 2 (a) shows the price time series of a simulation with a very small use *γ*. At any point in time, the system moves towards a certain price which characterises a local equilibrium. As the system approaches this equilibrium, price fluctuations are reduced. These fluctuations generally consist of complex oscillations like the one shown in Figure 2 (b). The equilibria become unstable once all predictable information is exploited by the speculators. Then, even little overshooting of the adaptation dynamics or noise can lead to price changes corresponding to information states that were not predicted by patterns in the immediate past. Because the market is not well-adapted to these new states, the possibility of large price changes increases dramatically. Compared to^18^, we here discovered an instability due to information annihilation in a mathematically different way, which demonstrates that this concept is even more general.

**Figure 3:**
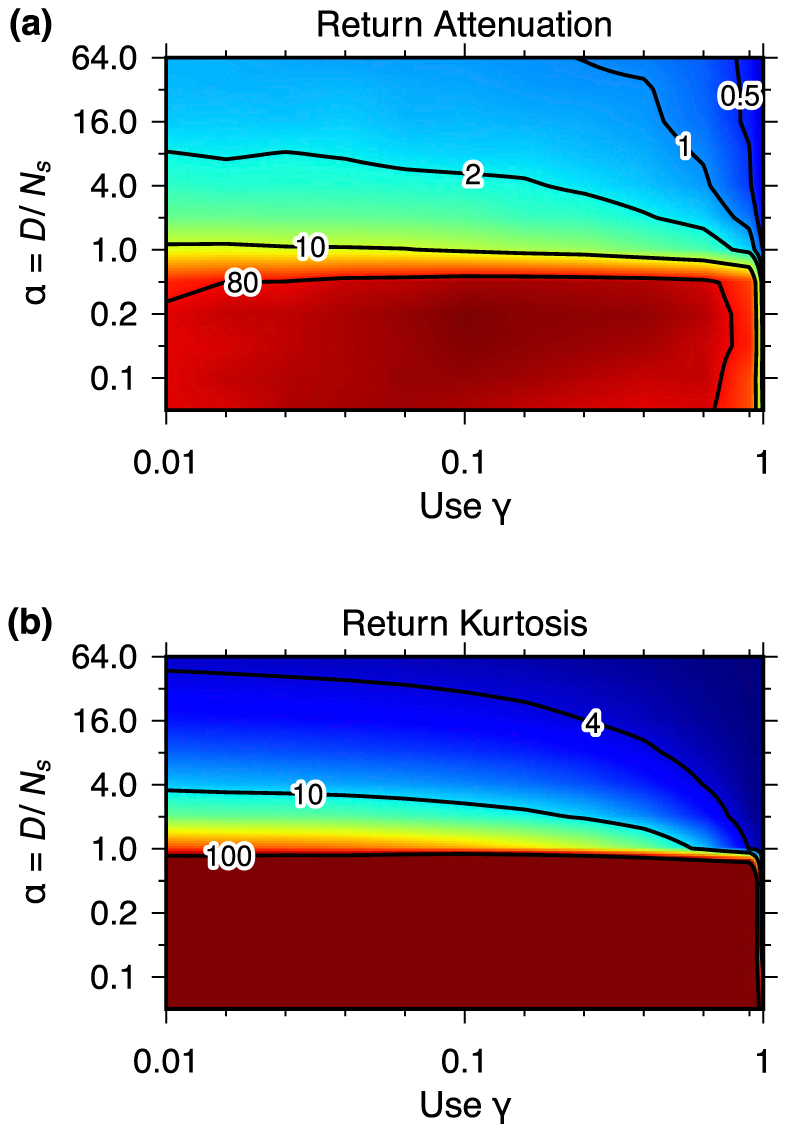
(a): reduction of average return magnitudes during transients and (b): kurtosis of log-returns of the model with endogenous information for different *α* vs *γ*. A kurtosis of 3 corresponds to a normal distribution. Here, the system size is set constant at *N_s_* = 2^10^ speculators and *N_p_* = 2^4^ producers. For each time series, 2 · 10^7^ time steps were simulated. Reductions are measured as the ratio between the mean log-return magnitudes during the first 10 and the last 10^7^ time steps. The kurtoses were calculated for the last 10^7^ time steps. Simulations were performed on a grid, all axis ticks correspond to node positions. For each node on the grid, 50 time series were analysed and results were averaged. Linear interpolation and colour mapping were performed after logarithmising the values at each node; contour line labels are the actual values for variances and kurtoses. For *α* ≤ 1/2, the kurtoses reach extreme values that can not be reliably estimated from finite time series. Therefore, the colour scale in (b) was set to not extend to values above 100.

For larger *γ*, this behaviour is not as obvious: time series appear random and distinct oscillations are rarely visually recognisable (Fig. 2 (c, d)). Still, phase diagrams from extensive simulations demonstrate that return distributions are largely unaffected by these effects over wide ranges of *γ*: Figure 3 (a) shows how much initial mean log return magnitudes are reduced for different *α* and *γ* and reflects the phase transition identified above. Figure 3 (b) shows the impacts that infrequent extreme returns have on the remaining variances, which are measured by kurtoses after transients. The stronger the reduction of return magnitudes (Fig. 3 (a)), the heavier tailed the return distributions are (Fig. 3 (b)). This establishes a clear link between information annihilation and extreme returns in our model for the whole parameter space, which is not found for exogenous information drawn from a uniform distribution (Fig. 1 (b), and supplement).

**Figure 4:**
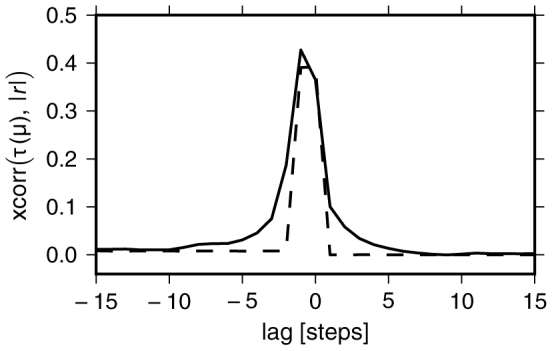
Correlation of return magnitudes |*r*| with the time *τ* since the corresponding information states occurred last. Model parameters: *D* = 2^10^, *N_s_* = 2^11^, *N_p_* = 0, *γ* = 0.5. Simulation length: *T* = 2 · 10^7^; the first *T*/2 time steps were discarded for the analysis. Black line: Endogenous information. Dashed line: Exogenous information with *P*_exo_(*μ*) ∝ exp(−0.02 *μ*), leading to *P*(*τ*) ∝ *τ*^−2^. Both lines are averages over 10 simulations.

We furthermore investigated if the log-return magnitudes can be quantitatively related to the novelty of the corresponding information states. Intuitively, states which did not occur in a long time are more surprising and therefore carry more information than the ones visited more recently – a concept that is closely related to Shannon information. We indeed find that large returns are caused by information states that have not occurred for a long time: The more surprising an information state is, the higher the corresponding log return. The correlation between log return magnitudes and the times *τ* that have passed since the respective information states occurred last is shown in Figure 4 for endogenous information (solid line). Here, the absorption of local information in combination with rare jumps leads to a strongly inhomogeneous distribution of visiting frequencies over the information set: the probability distribution *P*(*τ*) is power-law tailed with an exponent of approximately 2.5 (see supplement). This suggests that the self-reflexive dynamics for endogenous information generates a characteristic distribution of information states that ultimately underlies extreme price fluctuations. We tested this hypothesis by using inhomogeneously distributed exogenous information states that lead to similarly distributed *τ*. Then, as in the endogenous case, return magnitudes are strongly correlated with *τ* (Fig. 4, dashed line).

## Discussion

Our model demonstrates that a simple yet plausible mechanism of order size adaptation (namely the redistribution of assets via trading) suffices to coordinate the impacts of diverse strategies such that information becomes absorbed in the price. The model is therefore informationally efficient. When the same set of information states is repeatedly conveyed to the market, it quickly ceases to elicit any further price change. In fact, our results demonstrate that a market as a whole can learn in a manner comparable to what happens in the nervous system. As an analogy, imagine telling a joke that elicits strong laughter. Telling the same joke in the same way to the same listener shortly thereafter will presumably not lead to a similarly strong response.

The proof that the trading rules in our model correspond to an efficient gradient-based learning rule that minimises predictable return magnitudes provides a rigorous link of a fundamental market mechanism to adaptive control. We tested variations of this mechanism including adaptations of individual agent use parameters, and order-book based pricing^19^; information absorption proved to be a robust property. We therefore consider it highly plausible that it could prevail in real markets despite the complexities of real pricing mechanisms and order size adaptations, as long as the latter correlate with trading success.

In our model, “rationality” in the sense of an efficient adjustment of prices to new information emerges from the collective behaviour of many traders. This effect may be considered analogous to neuronal networks where cognitive functions are not present in single neurons, but emerge from their interaction.

We find that a minority rule with respect to the returns is a dominating factor in the stabilising part of the models dynamics. That is, traders whose actions counteract those of the majority profit most. In the well known minority game^17^ (MG), however, adaptation is based on the choices of the participating agents and a single-step payoff with respect to the excess demand. This differs substantially from our model, where the market adapts traders' impacts based on price changes and also over different time scales. Most importantly, the mechanism for herding previously discussed for MGs is a breakdown of the efficient coordination of agents in overcomplete markets where they become too correlated. This leads to an increase in average fluctuations and in sharp contrast to the model presented here, does not depend on how the information conveyed to the agents is generated.

In contradistinction, the heavy-tailed distributions here are a direct consequence of the elimination of local trends or patterns, which yields a net decrease in average fluctuation magnitudes. A general dynamic instability due to information annihilation by adaptive control can be realised independently of microscopic interactions^18^ and in fact was first realised in a one-dimensional random map^20^. These fluctuations are therefore a sign of high efficiency and do not signal its breakdown. Instead, they reflect surprising information to which the system is the more susceptible the better it is locally adapted. This finding exactly reflects one aspect of the efficient market hypothesis, namely that only new information leads to price changes. An important new finding in our model is that endogenously generated news can account for the extreme price fluctuations that were not caused by external news. This distinct role of endogenous information is not found in common minority games. Therefore, the results presented in this paper are at least complementary to the published findings based on MGs in several respects. It is, however, possible to formulate a minority game corresponding to our model, but that would go beyond the scope of this paper.

Our finding that efficient information annihilation does not result in a unique and stable equilibrium, but instead can lead to local states that perpetually become unstable, provides a new and comprehensive solution to a long-standing antinomy in economics where both notions have been considered mutually exclusive^16^. Our work also demonstrates that the view of markets as complex dynamical systems is not per se in contradiction with existing economic concepts. Instead, a more complete economic theory may combine aspects of both paradigms. Since critical information annihilation was demonstrated in normative behavioural models subjected to constraints^18^,^20^, we cannot rule out that new types of representative agent models might also display such properties. This opens up the possibility of a consistent transition between microscopic and macroscopic models, as found in many physical theories.

While our trading model might be considered a new instance of self-organised criticality^21^, the underlying principle is distinct from previous instantiations since it also occurs in low-dimensional systems^20^. Furthermore, our adaptive agent-based model not only demonstrates the theoretical principle of information annihilation instability, but can also reproduce important “stylised facts” of financial markets quantitatively to a surprising extent. The parameter sets that best reproduce the data correspond to situations where agents on average profit most (see supplement). This is plausible because real traders can choose among different markets. These findings suggest that the information annihilation instability is indeed one of the main causes of the notorious large jumps in real price time series, particularly in strongly speculative markets. An empirical confirmation of our theory would require identifying the information states that cause large price changes in a given market, for which it then makes directly testable predictions.

## Author Contributions

F.P. and K.P. wrote the manuscript and performed the analytical calculations together. F.P. performed the simulations and prepared the figures shown in the manuscript while K.P. performed additional simulations whose results are not shown.

## Additional information

**Data sources**: Data from: Department of Statistics at Carnegie Mellon University; download: http://www.analyzeindices.com/dowhistory/djia-100.txt.

## Supplementary Material

Supplementary Information
