# Supplementary material for: An Inherent Instability of Efficient Markets
Source: Sci Rep. 2013 Sep 27;3:2784. doi: 10.1038/srep02784 (PMC3784944; doi:10.1038/srep02784)
Supplement: Supplementary Information [file srep02784-s1.pdf]

Supplementary Materials for

An Inherent Instability of Efficient Markets

Felix Patzelt and Klaus Pawelzik

correspondence to felix@neuro.uni-bremen.de

Contents

I. Supplementary Methods 2

A. Model 2

B. Invariant Manifold 4

C. Completeness of the Strategies 4

D. Gradient Descent 8

II. Supplementary Figures 11

A. Phase Diagrams for Exogenous Information 11

B. Speculators and Producers 12

C. Distribution of Information Ages (Surprise) 13

D. Mixed Information 14

III. Supplementary Discussion 15

A. Income and the Critical Point 15

B. Volatility and distribution tails 17

References 19

I. SUPPLEMENTARY METHODS

A. Model

Each agent $i = 1, \ldots, N$ possesses two types of assets which without loss of generality are called money $M_i(t)$ and stocks $S_i(t)$. N is the total number of agents. In each round, every agent places a market order to either buy or to sell an amount of stocks. Since trading should conserve the amount of traded assets, the price in each round is determined by:

$$p(t) = \frac{\delta(t)}{\varsigma(t)} \tag{1}$$

with demand δ and supply of stocks ς. This is a fair rule that could be used in a real market with only market orders executed at distinct points in time.

For a market including stochastic limit orders gathered over some period of time, consider the hypothetical price $p^*(t)$ at which trades would take place if all agents scaled their orders by a common factor. Then the volume would change, but to preserve market clearing the price is not affected; that is $p(t)^* = p(t)$. Therefore, the price is a function of the ratio of demand and supply. After linearisation of this function for small small excess demands the price is proportional to the aforementioned ratio which justifies this choice of the pricing rule also as an approximation of the mean prices obtained from limit orders.

Agents base their decisions on a public information state. In each time step one of D possible states, which is denoted by an index $\mu(t)$, is conveyed to the agents. We distinguish two different methods for the generation of these information states at each time step t:

For exogenous information, $\mu(t)$ are independent identically distributed random variates drawn from a distribution $P_{\text{ext}}(\mu)$.

For endogenous information, agents possess a memory of the most recent K signs of the log returns r. To eliminate the possibility of a lock, signs for vanishing returns are chosen at random. This information can take one out of $D = 2^K$ possible states:

$$\mu(t) = \sum_{i=0}^{K-1} 2^i \, \Theta\big(r(t-i-1) + \eta(t-i-1)\big) + 1 \tag{2}$$

where Θ is the step function, and η is an arbitrarily small symmetric random variable with zero mean. Simulation results do not depend on $\text{Var}(\eta)$ as long as it is small enough.

Each agent's i consequent decision is now determined by a strategy vector whose elements σ_i^μ initially are drawn randomly out of $\{0, 1\}$ and then kept constant. These two possible de-

cisions correspond to trading an amount $m_i(t)$ of money or an amount $s_i(t)$ of stocks for the respective other asset in the next round. Orders are placed with a constant use parameter γ:

Case $\sigma_i^{\mu(t)} = 1$ (agent i buys stocks):

$$\begin{aligned} m_i(t) &= \gamma\, M_i(t) \\ s_i(t) &= 0 \end{aligned} \tag{3}$$

Case $\sigma_i^{\mu(t)} = 0$ (agent i sells stocks):

$$\begin{aligned} m_i(t) &= 0 \\ s_i(t) &= \gamma\, S_i(t) \end{aligned} \tag{4}$$

Demand and supply are the sums of all buy and sell orders respectively

$$\delta(t) = \sum_{i=1}^{N} m_i(t) + \epsilon \tag{5}$$

$$\varsigma(t) = \sum_{i=1}^{N} s_i(t) + \epsilon \tag{6}$$

where ϵ is a small positive number. This ensures that prices and returns are always well defined. The cases with zero demand or supply are, however, irrelevant for all practical purposes. A sufficiently small $\epsilon \ll 10^{-3}$ does not influence simulation results to a meaningful degree. All figures were generated using $\epsilon = 10^{-10}$.

We investigate the effect of a market ecology by dividing the agents into N_s speculators and $N_p = N - N_s$ producers. We focus on markets that are dominated by speculators whose resources are redistributed due to trading:

$$\left.\begin{aligned} M_k(t+1) &= M_k(t) - m_k(t) + s_k(t)\, p(t) \\ S_k(t+1) &= S_k(t) - s_k(t) + m_k(t)/p(t) \end{aligned}\right\} N_p < k <= N. \tag{7}$$

Producers' resources stay constant throughout the game:

$$\left.\begin{aligned} M_j(t) &= M_j(0) \\ S_j(t) &= S_j(0) \end{aligned}\right\} \quad j < N_p. \tag{8}$$

Thus, only speculators redistribute their wealth and are competitive whereas producers provide a predictable supply of liquidity and stocks. All agents are initially provided with equal amounts of resources $M_i(0) = S_i(0) = 1$.

B. Invariant Manifold

We show, that if one distribution of resources $(\overline{M}, \overline{S}) = (\overline{M}_1, \ldots, \overline{M}_N, \overline{S}_1, \ldots, \overline{S}_N)$ exists for which the price $p(\overline{M}, \overline{S}, \mu) = \overline{p}$ is independent of the information μ, this price is invariant with respect to any resource redistribution due to trading in a purely speculative market. That is, there is a manifold $Q = \{(\overline{M}', \overline{S}') \mid p(\overline{M}', \overline{S}', \mu) = \overline{p} \; \forall \; \mu\}$ of distributions of stocks for which the price is independent of μ and this manifold is closed with respect to trading according to Eq. (7). For the proof, assume that at some point in time the system is in a suitable state such that

$$\frac{\delta(\overline{M}, \mu)}{\varsigma(\overline{S}, \mu)} = \overline{p} \quad \forall \; \mu(t) \Leftrightarrow \tag{9}$$

$$\begin{aligned} \delta(\overline{M}, \mu) - \overline{p}\, \varsigma(\overline{S}, \mu) &= \\ \gamma \sum_{i=1}^{N} \left(\sigma_i^{\mu} \, \overline{M}_i - \overline{p} \, (1 - \sigma_i^{\mu}) \, \overline{S}_i \right) &= 0 \quad \forall \; \mu(t). \end{aligned} \tag{10}$$

Then, denoting the distributions of stocks and money after trading by $\overline{M}_i'$ and $\overline{S}_i'$ we obtain:

$$\frac{1}{\gamma} \left(\delta(\overline{M}', \mu') - \overline{p}\, \varsigma(\overline{S}', \mu') \right) = \sum_{i=1}^{N} \sigma_i^{\mu'} \, \overline{M}_i' - \overline{p} \sum_{i=1}^{N} (1 - \sigma_i^{\mu'}) \, \overline{S}_i' \tag{11}$$

$$\begin{aligned} = & \sum_{i=1}^{N} \sigma_i^{\mu'} \, \left(\overline{M}_i - \gamma \, \sigma_i^{\mu} \, \overline{M}_i + \gamma \, \overline{p} \, (1 - \sigma_i^{\mu}) \, \overline{S}_i \right) \\ & - \overline{p} \sum_{i=1}^{N} (1 - \sigma_i^{\mu'}) \left(\overline{S}_i - \gamma \, (1 - \sigma_i^{\mu}) \, \overline{S}_i + \frac{\gamma}{\overline{p}} \, \sigma_i^{\mu} \, \overline{M}_i \right) \end{aligned} \tag{12}$$

$$= \sum_{i=1}^{N} \left(\sigma_i^{\mu'} \, \overline{M}_i - \overline{p} \, (1 - \sigma_i^{\mu'}) \, \overline{S}_i \right) - \gamma \sum_{i=1}^{N} \left(\sigma_i^{\mu} \, \overline{M}_i - \overline{p} \, (1 - \sigma_i^{\mu}) \, \overline{S}_i \right) \tag{13}$$

$$= 0 - 0 = 0 \tag{14}$$

□

C. Completeness of the Strategies

As shown above, a sufficient condition for complete suppression of all price changes is finding a resource distribution $(\overline{M}, \overline{S})$ for which the price is independent of the information. That is,

$$p(\mu, \overline{M}, \overline{S}) = \overline{p} \; \forall \; \mu \tag{15}$$

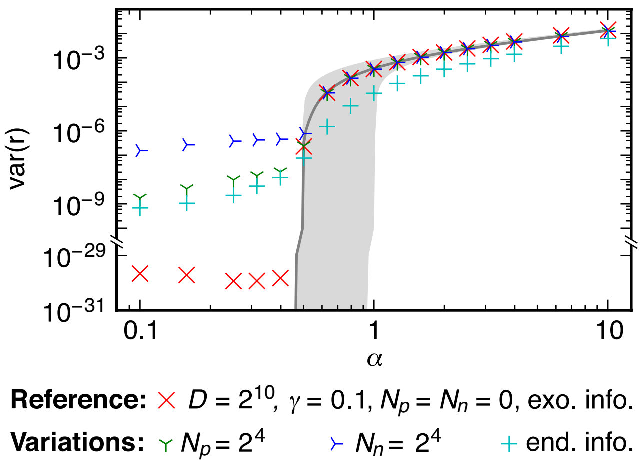

FIG. S1: Average log-return variances for different values of $\alpha = D/N_s$. The model with exogenous information and only speculators serves as a reference. For comparison, simulations with a small numbers of either deterministic ($N_p = 2^4$) or random producers ($N_n = 2^4$) are shown as well as as the model with endogenous information. Grey area: Analytical upper and lower limit for exogenous information. Dark grey line: heuristic interpolation.

which is equivalent to Eq. (10). To fulfil this criterion, we need enough agents to form a complete basis in the strategy space which has D dimensions. Then, the deviation from $\overline{p}$ caused by each agent can be cancelled by a superposition of the other agents for every μ. This can be guaranteed if the number of speculators N_s exceeds $2D$.

For an insufficient number of speculators, we can still calculate an upper and a lower bound for the variance of the log returns given D and N for a perfect superposition of speculators with exogenous information. Numerical and analytical results for this case are shown in Fig. S1. The mean variance is found to drop dramatically at $\alpha = D/N_s = 1/2$, with an increasingly sharp transition for large D. This phase transition can be understood by considering the probability that a random binary vector can be cancelled by an optimal superposition of $N - 1$ random binary vectors with positive weights. As an interim step, consider superpositions of random vectors with arbitrary weights. One such vector creates a one dimensional subspace. Adding a second vector expands the dimensionality of the subspace to $d_2 = 2$ if it is linearly independent of the first one. Adding further vectors one by one, the probability that the ith vector does not lie in a d_{i-1}-dimensional submanifold is

$$P(d_i = d_{i-1} + 1) = 1 - 2^{D - d_{i-1}}. \tag{16}$$

We can therefore iteratively calculate the probability distribution $P(d_{N_s-1})$ of d after adding $N_s - 1$ agents and the probability

$$P(d_{N_s} = d_{N_s-1} + 1) = \sum_{d=1}^{D} P(d = d_{N_s-1})\ (1 - 2^{d-D}) \tag{17}$$

that one out of N_s agents is linearly independent of the others. If a vector is linearly independent of the other agents in d dimensions, it cannot be cancelled by a linear combination of the other agents for all μ. However, it may still be possible to cancel this agent's impact for a subset of all possible μ, i.e. for a smaller subspace. Therefore, the probability that an agent cannot be cancelled in any given time step is

$$P_{\text{c.c.}} = \sum_{d=1}^{D} P(d = d_{N_s-1})\ (1 - 2^{d-D})\ (1 - \frac{d}{D}). \tag{18}$$

The last term weights each summand with the fraction of dimensions in which the agent's impact is not cancelled. Finally, to relate the fraction of not cancelled agents to returns we need to consider the fluctuations prior to any resource redistribution. Since all strategies and μ are chosen randomly, agents initially contribute to the demand or the supply at random. These fluctuations of demand and supply then follow a binomial distribution with N_s trials and equal probability for buying or selling:

$$\delta \propto \mathcal{B}(N_s, 1/2) \tag{19}$$

$$\varsigma \propto \mathcal{B}(N_s, 1/2). \tag{20}$$

Since

$$\langle\delta\rangle = \langle\varsigma\rangle = N_s/2, \quad \text{and} \tag{21}$$

$$\text{Var}(\delta) = \text{Var}(\varsigma) = N_s/4 \tag{22}$$

we can approximate the price for small deviations:

$$p = \frac{N/2 + \Delta\delta}{N/2 - \Delta\varsigma} \approx 1 + 2\frac{\Delta\delta - \Delta\varsigma}{N_s}. \tag{23}$$

Therefore,

$$\langle p(0)\rangle \ = \ 1, \tag{24}$$

$$\text{Var}(p(0)) \ \approx \ 4/N_s, \tag{25}$$

and finally

$$\langle r(0)\rangle = 0, \tag{26}$$

$$\mathrm{Var}(r(0)) \approx \frac{8}{N_s \ln(10)^2}, \tag{27}$$

where the logarithm stems from using the logarithm with base 10 in the log return.

Combining Eqns. (18) and (27), we obtain the expected variance of the return for an optimal superposition of agents without the positivity constraint on the resources

$$\mathrm{Var}(r_{\text{n.c.}}) = \mathrm{Var}(r(0))\ P_{\text{c.c.}}. \tag{28}$$

Since resources cannot be negative, they form a positive cone. Each agent that is linear independent of the others spans a half space. Therefore, $2N_s$ agents are necessary to completely span the strategy space. Yet for small numbers of agents, each agent still represents a full degree of freedom since the probability that two agents lie on the same 1-dimensional submanifold is vanishingly small. However, as the number of agents is increased such that $\alpha \to 1$, an increasingly large number of new agents just converts a halfspace into a full one. Therefore, Eq. (28) represents a lower limit for the variance of the log returns which is a good description for $N_s \ll D$. An upper limit is obtained by changing Eq. (18) such, that each agents increases d by 1/2. This is a good approximation for $N_s \approx 2D$. The area in between these limits is shown in Fig. S1 (grey shaded). The lower limit has a phase transition at $\alpha = 1$ while the upper limit has a phase transition at $\alpha = 1/2$. A phase transition at $\alpha = 1$ is already present in Eq. (17). The gradual convergence for the true variance of the system from the lower to the upper limit is captured by a simple heuristic interpolation: For the dark grey line in Fig. S1, the probability for a new linearly independent agent to increase d by one is $P_1 = \min(1, N_s/2^{m+1})$ while the probability to increase d by 1/2 is $P_{1/2} = 1 - p_1$. The presented theory describes the numerical results (Fig. S1) for the model with endogenous information very well for $\alpha \leq 1/2$. For full markets, the residual error for simulations with only speculators is determined by the numerical precision. When producers are present, the residual error is noticeably higher. This is due to the fact, that producers push the system off the invariant manifold. This error is dependent on the agents' use and vanishes for small γ. Still, predictable producers are cancelled much better than random ones because speculators can successfully predict their choices. For endogenous information ($D = 2^K$), the phase transition appears smother and slightly shifted towards larger

α. A stronger reduction of average returns for $\alpha < 1/2$ occurs due to the more localised adaptation: Agents do not adapt to all possible values of μ at the same time.

D. Gradient Descent

We now investigate, how the system evolves towards the invariant manifold. We focus on large numbers of agents and small γ. The resource redistribution due to subsequently trading the two assets for one another is found to be a special case of a learning rule which minimises log-return magnitudes. Even more generally, we consider the error function

$$e = r^2 \tag{29}$$

and show that its gradient

$$\frac{\partial e}{\partial X} = 2r\frac{\partial r}{\partial X}, \quad X \in \{M_1, \ldots M_N, S_1, \ldots, S_N\} \tag{30}$$

with respect to the agents' resources is dominated by terms with the opposite sign as the change in the agents' resources. Therefore, any scaling of the agents' resources which keeps the sign of the return for money and the opposite sign for stocks corresponds to minimising log return magnitudes similar to a gradient descent.

To begin with, consider two subsequent time steps where the information takes the states denoted by μ and μ' respectively. We again consider a market consisting of speculators only. The derivative of the return with respect to the resources of an agent k is

$$\frac{\partial r(M,S,\mu,\mu')}{\partial M_k} = \frac{\sigma_k^{\mu'}}{\delta'} - \frac{\sigma_k^{\mu}}{\delta} + O(\gamma), \tag{31}$$

$$\frac{\partial r(M,S,\mu,\mu')}{\partial S_k} = \frac{1-\sigma_k^{\mu}}{\varsigma} - \frac{1-\sigma_k^{\mu'}}{\varsigma'} + O(\gamma), \quad \text{with} \tag{32}$$

$$\delta = \delta(M,\mu), \qquad \delta' = \delta(M',\mu'), \tag{33}$$

$$\varsigma = \varsigma(S,\mu), \qquad \varsigma' = \varsigma(S',\mu'). \tag{34}$$

The change in resources after trading twice is

$$\Delta M_k = M_k'' - M_k = \gamma\left(S_k((1-\sigma_k^{\mu})p + (1-\sigma_k^{\mu'})p') - M_k(\sigma_k^{\mu'} + \sigma_k^{\mu})\right) + O(\gamma^2) \tag{35}$$

$$\Delta S_k = S_k'' - S_k = \gamma\left(M_k\left(\frac{\sigma_k^{\mu'}}{p'} + \frac{\sigma_k^{\mu}}{p}\right) - S_k(2 - \sigma_k^{\mu} - \sigma_k^{\mu'})\right) + O(\gamma^2). \tag{36}$$

We are interested in

$$\Delta r_k = \left(\Delta M_k \frac{\partial r}{\partial M_k} + \Delta S_k \frac{\partial r}{\partial S_k}\right) \tag{37}$$

and continue only with leading terms in γ.

For now, we also assume that agents can only perform roundtrip trades (RT). The general case will be discussed later. Two cases are left:
Case $\sigma_k^{\mu}(1-\sigma_k^{\mu'}) = 1$:

$$\frac{\Delta r_k^{RT}}{\gamma} \overset{\gamma\ll 1}{\approx} \frac{M_k - p'S_k}{\delta} - \frac{M_k/p - S_k}{\varsigma'} \tag{38}$$

$$= \frac{M_k}{\delta}(1-\frac{\varsigma}{\varsigma'}) + \frac{S_k}{\varsigma'}(1-\frac{\delta'}{\delta}) \tag{39}$$

Case $\sigma_k^{\mu'}(1-\sigma_k^{\mu}) = 1$:

$$\frac{\Delta r_k^{RT}}{\gamma} \overset{\gamma\ll 1}{\approx} \frac{pS_k - M_k}{\delta'} + \frac{M_k/p' - S_k}{\varsigma'} \tag{40}$$

$$= \frac{M_k}{\delta'}(\frac{\varsigma'}{\varsigma} - 1) + \frac{S_k}{\varsigma}(\frac{\delta}{\delta'} - 1). \tag{41}$$

Above, we used $p = \delta/\varsigma$ and $p' = \delta'/\varsigma'$. Then,

$$\frac{1}{\gamma}\sum_{k=1}^{N} \Delta r_k^{RT} = \frac{\varsigma'}{\varsigma} - \frac{\varsigma}{\varsigma'} + \frac{\delta}{\delta'} - \frac{\delta'}{\delta} \tag{42}$$

$$= \frac{\varsigma'}{\varsigma}(1-\frac{p'}{p}) + \frac{\varsigma}{\varsigma'}(\frac{p}{p'} - 1) \quad \begin{cases} < 0, & r > 0 \\ > 0, & r < 0 \end{cases} . \tag{43}$$

Therefore, the change in the total error function

$$\sum_{k=1}^{N} \left(\Delta M_k \frac{\partial r^2}{\partial M_k} + \Delta S_k \frac{\partial r^2}{\partial S_k}\right) \overset{\mathrm{RT}}{\leq} 0 \tag{44}$$

can never be positive if agents only perform roundtrip trades.

On average, this result holds even for the general case. The reason why we have to consider averages is, that agents who buy or sell two times in a row always decrease the amount of money or stocks they own after two time steps. Therefore, these agents' resources are expected to change in the opposite direction of the gradient half of the time. That is, for every given pair of informations (μ, μ'), a quarter of all agents' is expected to have their resources evolve such that future $r(\mu, \mu')^2$ increase. However, the actual influence of these agents is much lower and can be neglected for large systems. Then, demand and supply can

be well described as binomial processes as shown above. Here, express demand and supply as:

$$\delta = \tfrac{N}{2} + \Delta\delta, \quad \langle \Delta\delta \rangle = 0, \quad \langle \Delta\delta^2 \rangle \le \frac{N}{4} \tag{45}$$

$$\varsigma = \tfrac{N}{2} + \Delta\varsigma, \quad \langle \Delta\varsigma \rangle = 0, \quad \langle \Delta\varsigma^2 \rangle \le \frac{N}{4} \tag{46}$$

and the relative fluctuations around the mean demand $N/2$ are only $\sqrt{N}/2$ and therefore small for large N. Thus we can expand Eq. (31) for small fluctuations:

$$\frac{\partial r(M,S,\mu,\mu')}{\partial M_k} \approx \sigma_k^{\mu'}\left(\frac{2}{N} - \frac{4\Delta\delta'}{N^2}\right) - \sigma_k^{\mu}\left(\frac{2}{N} - \frac{4\Delta\delta}{N^2}\right) \tag{47}$$

$$\frac{\partial r(M,S,\mu,\mu')}{\partial S_k} \approx (1-\sigma_k^{\mu})\left(\frac{2}{N} - \frac{4\Delta\varsigma}{N^2}\right) - (1-\sigma_k^{\mu'})\left(\frac{2}{N} - \frac{4\Delta\varsigma'}{N^2}\right). \tag{48}$$

As the above equation shows, when agents perform roundtrip trades, they contribute a term of order N^{-1} to the gradient with respect to each asset. When agents buy or sell twice, they only contribute a term of order $N^{-1.5}$ for one asset. Therefore, the influence of these agents vanishes for sufficiently large N. By a similar argument, approximately a quarter of all agents performs either one of the actions (buy, sell), (sell, buy), (buy, buy), and (sell, sell) while fluctuations can be neglected for large N. Summing up, the expected change in $r(\mu,\mu')^2$ over repeated trades with the same information

$$\left\langle \sum_{k=1}^{N}\left(\Delta M_k \frac{\partial r^2}{\partial M_k} + \Delta S_k \frac{\partial r^2}{\partial S_k}\right)\right\rangle_{(\mu,\mu')} \le 0. \tag{49}$$

is always negative given a sufficiently large number of agents.

II. SUPPLEMENTARY FIGURES

A. Phase Diagrams for Exogenous Information

For uniformly distributed exogenous information states, a reduction of fluctuations does not generally increase the kurtosis (Fig. S2). This is opposed to the endogenous case shown in Fig. 3 in the main paper. Only very large uses in overcomplete markets cause high kurtoses. Then, even uniformly distributed μ occasionally are not repeated for a sufficiently long time to be "forgotten" by the market.

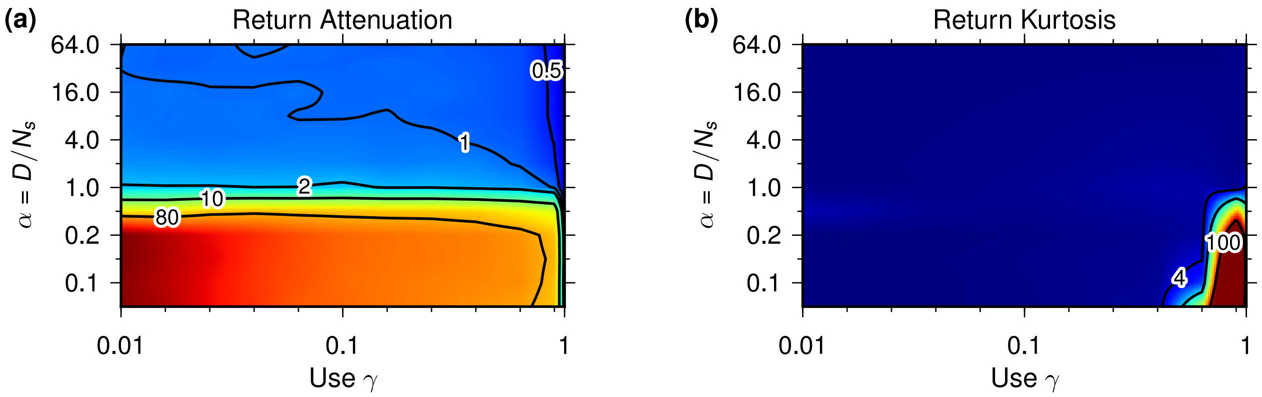

FIG. S2: **(a)**: Reduction of average return magnitudes during transients, and **(b):** kurtoses for the model with exogenous μ drawn from a uniform distribution. All other parameters and figure generation are identical to Fig. 3 in the main paper.

B. Speculators and Producers

Fig. S3 shows the phase diagram for α versus the amount of producers in the market. As it turns out, a second phase transition with respect to the number of producers is found. This transition is independent of the one for the speculators. Small $N_p < 0.5 \cdot D$ only weakly influence return distributions.

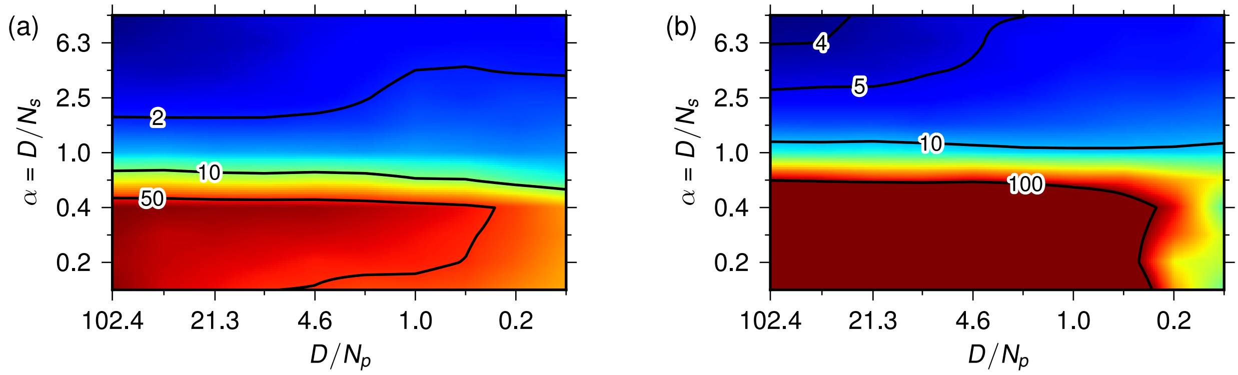

FIG. S3: **(a)**: Reduction of average return magnitudes during transients, and **(b):** kurtosis of log-returns of the model with endogenous information for different numbers of speculators N_s and producers N_p for constant memory $K =$ 10 (i.e. $D = 2^{10}$) and use $\gamma = 0.5$.

C. Distribution of Information Ages (Surprise)

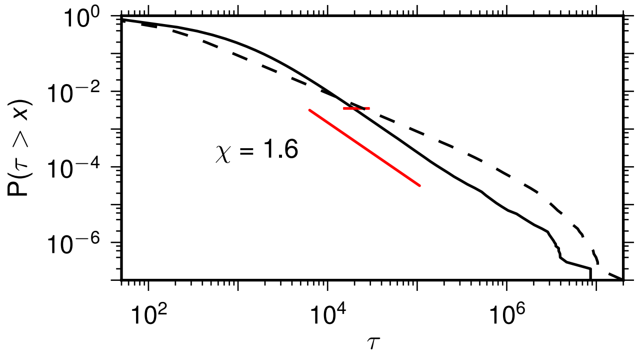

FIG. S4: Complementary cumulative distribution of the times $\tau(t)$ since the informations $\mu(t)$ occurred last. Solid black line: Model with intrinsic information and $D = 2^{10}$, $N_s = 2^{11}$, $N_p = 0$, $\gamma = 0.5$. Short red line: Hill estimator. Dashed line: Exogenous information with $P_{\rm exo}(\mu) \propto \exp(-0.02\,\mu)$, leading to $P(\tau) \propto \tau^{-2}$.

D. Mixed Information

For a combination of endo- and exogenous information, results are similar to pure endogenous information as long as the endogenous part dominates. Generally, more exogenous information leads to a stronger reduction of fluctuations, less pronounced volatility clustering, and random time series without visible patterns even for small γ. The scaling of the remaining extreme returns remains unchanged. An example is shown in Fig. S5.

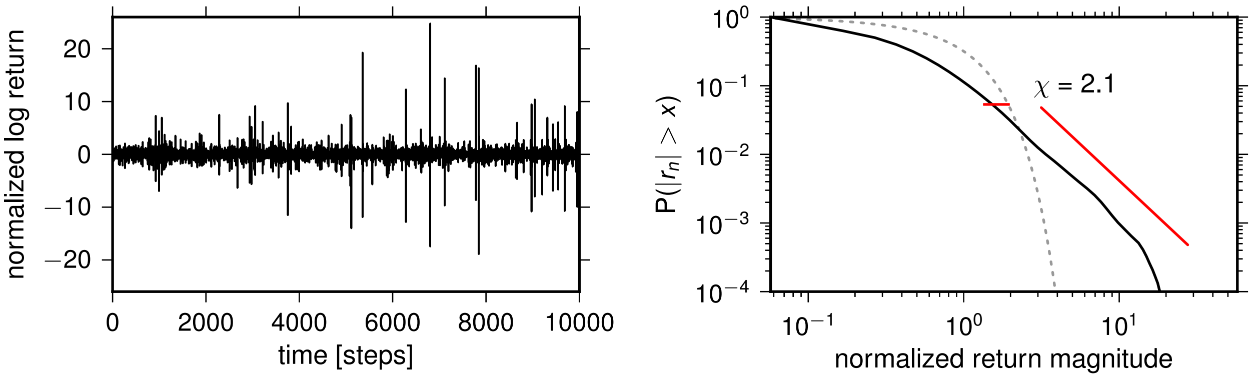

FIG. S5: Simulation for a model with 3 bits of uniform exogenous information and 6 bits of endogenous information. $N_s = 2^{10}$, $N_p = 0$, $\gamma = 0.1$. The first 2^7 time steps were discarded. Log returns r_n are normalised by their standard deviation. **(a)**: Time series **(b):** Solid black line: complementary cumulative distribution function. Short Red line: Hill estimator for the scaling exponent. Dashed grey line: normal distribution.

III. SUPPLEMENTARY DISCUSSION

A. Income and the Critical Point

Fig. S6 shows the phase transition with respect to α in more detail. As in Fig. S1, we take one parameter set as a reference to which we compare simulations after transients for different parameters. For orientation, the log-return variances (Fig. S6 (a)) and kurtoses(Fig. S6 (b)), which have been discussed earlier, are shown again.

Mean speculator capitals

$$C_s(t) = \frac{1}{2N_s} \sum_{k=1}^{N_s} M_k(t) + S_k(t). \tag{50}$$

are not constant over time in markets that include producers. For empty markets, the ratio of average speculator and producer capitals quickly evolves towards an equilibrium. The more agents are added, the longer it takes for C_s to saturate. For critical or crowded markets a positive speculator income persists over long times. Then, average speculator capitals after transients can be well described as:

$$C_s(t)^2 = C_s(t_0)^2 + a\,t, \quad t_0 < t. \tag{51}$$

The income factor a is shown in Fig. S6 (c) and quantifies how well the speculators can exploit the producers. a is found to be independent of the initial ratio between speculator and producer capitals. It becomes maximal close to the critical point which can be intuitively understood: For empty markets, there is a finite chance for a producer strategy to lie outside of the space spanned by the speculators. Therefore, increasing the number of speculators increases their average income. For crowded markets, producers are already optimally exploited. Then, adding more speculators just distributes the maximal total income over more of them. An analogous maximum can be found in Minority Games (see e.g. ref. 15 in the main paper).

Fig. S6 (d) shows the Gini index, a common measure of wealth inequality. Increased incomes coincide with increased capital inequality among speculators as the gini-index shows a maximum at the critical point. Thus, only few speculators are most successful in exploiting the producers.

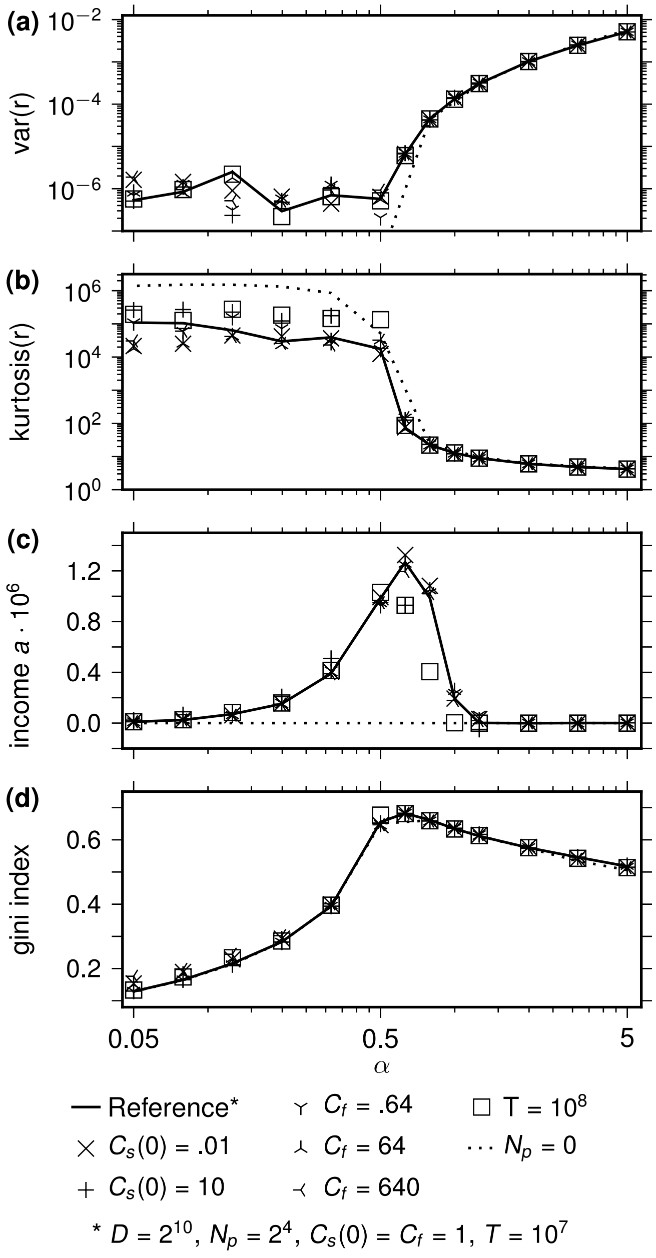

FIG. S6: Properties of log-return and resource distributions depend on the parameter $\alpha = D/N_s$. Solid line: The model with unity (initial) speculator and producer capitals C_s and C_p and a small number of producers serves as a reference. Shown are averages from 50 simulations for each of which only the last $T = 10^7$ out of $2\,T$ time steps have been analysed. **(a)**: variances and **(b)**: kurtoses were calculated from log-returns. **(c)**: income factors a according to Eqns. (50) and (51). **(d)** gini indices for speculator capitals after $2\,T$ time steps. For each other line or symbol, only one model parameters has been changed, respectively.

B. Volatility and distribution tails

From a purely descriptive point of view, the distribution and autocorrelation of a random process are mathematically distinct features. For example, two processes can have the same probability distribution, but different autocorrelations. However, there are various ways of generating a random process where these two features are closely interdependent. Log returns in particular are sums over many logarithmic price changes. Therefore, if those price changes were independent and identically distributed it follows from the generalised central limit theorem that returns over longer time intervals should follow either a Gaussian or a Lévy stable distribution. Yet, returns have been found to be outside of Lévy regime and still non-Gaussian, as well as remarkably stable as time intervals are increased[1,2]. Since subsequent price magnitudes are not independent, it seems natural to assume that a dynamic volatility is to blame for the slow convergence of long-term returns towards the Gaussian distribution. A possibly related finding is that return distributions become less heavy tailed after normalisation by an estimate of the volatility at each point in time. The extend of this effect depends on the assumptions made about a hidden stochastic volatility process[3,4].

In our model, jumps to a "surprising" part of the information (attractor) space creates large returns. Such events increase the probability for more large jumps while the market adapts to the new environment. Therefore, volatility in our model is a stochastic variable, and connected to non-Gaussian returns, but this connection is more complex than simply Gaussian noise with a time dependent amplitude. However, a detailed analytical characterisation of this volatility process is outside of the scope of this work. Here, we present two numerical analyses of the model returns.

First, distributions for the model are very stable when returns over more than one time step are calculated. Fig. S7 shows a comparison of single-step returns and cumulated returns over 1000 time steps for the model, and for similarly distributed independent random variates. The return distributions for the model are found to be more stable than the independent ones. Therefore, one time step in the model could also be interpreted as a shorter time interval than one day. This holds especially when increasing N_s which leads to a slower decay of correlations of magnitudes over time. For a comparable analysis of high frequency returns see, e.g.,[1].

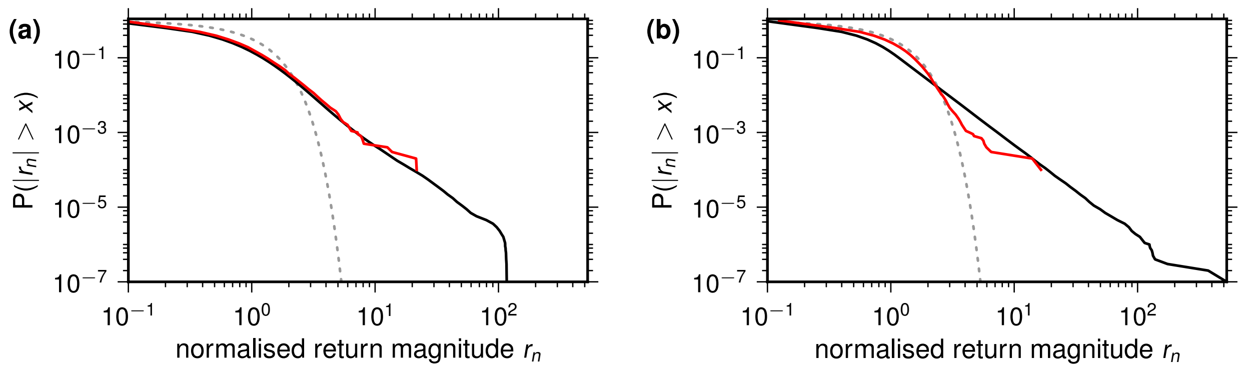

FIG. S7: Comparison of log returns (black line) and cumulated log returns over 1000 time steps calculated from the same time series (red line). In both cases, the distributions have been normalised to unit variance. A normal distribution (dashed grey line) is shown for comparison. **(a)**: Log returns for the model with $D = 2^{11}, N_s = 2^{12}$, $N_p = 0$, $\gamma = 0.8$. Transients were discarded. **(b)**: As surrogate log returns (black line), independent random variates were generated using inverse transform sampling. The cdf was chosen such that it follows a Gaussian below a threshold and a power law with a slope of 2.5 above the threshold. At the threshold, the cdf has a continuous derivative.

Second, we consider a simple analysis for how the cdf and local volatility interact. Returns are normalised by using sliding windows of different sizes (similar to[3]). The model behaves very similar to the Dow-Jones in this analysis (Fig. S8): Normalisation to constant estimated volatility reduces the kurtosis, but not enough to yield a Gaussian process. This effect is not found for independent surrogate returns.

Concluding, simple model-free tests show that the model captures observed features of volatility clustering in real stock market returns very well, and that simulated time steps do not necessarily have to be interpreted as trading days.

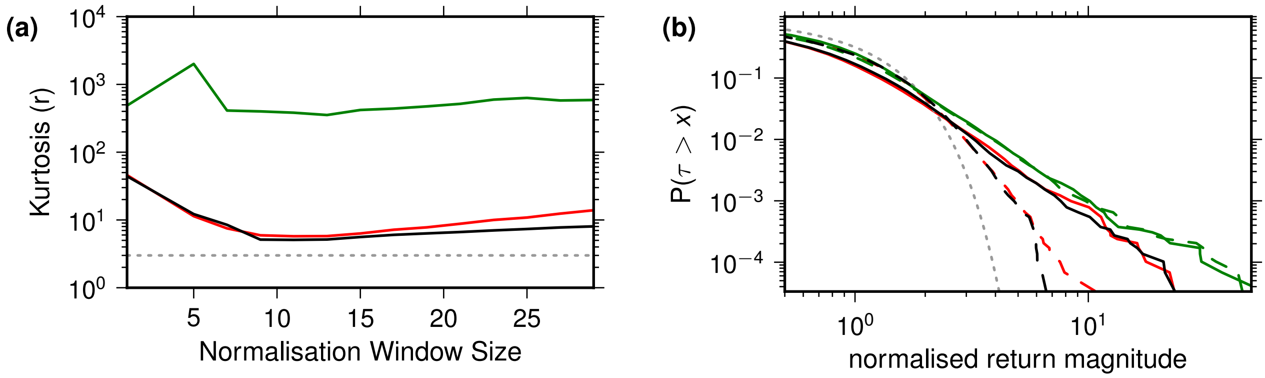

FIG. S8: Normalisation of log returns with a sliding window reduces the heavy distribution tails for the model (red lines, $D = 2^9, N_s = 2^{10}$, $N_p = 0$, $\gamma = 0.8$), and for the dow jones (black lines). The reduction is not sufficient to make the distribution perfectly Gaussian (dotted grey lines). For independent surrogate returns generated as in Fig. S7, the normalisation has no effect (green lines). **(a)**: Kurtosis of the log returns for different sizes of the normalisation window. **(b)**: Cdfs for the original (solid lines), and normalised (dashed lines) returns for the window sizes which minimise the respective kurtoses. All distributions are normalised to unit variance independently of the sliding normalisation to unit volatility.

---

1. Gopikrishnana, P., Pleroua, V., Liua, Y., Amarala, L., Gabaix, X. & Stanley, H. Scaling and correlation in financial time series. *Physica A* **287**, 362–373 (2000).
2. Lux, T. Stochastic Behavioral Asset-Pricing Models and the Stylized Facts. In *Handbook of Financial Markets: Dynamics and Evolution* (eds. Hens, T. & Schenk-Hoppe, K. R.) (North-Holland, Elsevier, 2009).
3. Andersen, T. G., Bollerslev, T., Diebold, F. X. & Labys, P. Exchange Rate Returns Standardized by Realized Volatility are (Nearly) Gaussian. *Multinational Finance Journal* **4**, 159–179 (2000).
4. Fuentes, M. A., Gerig, A. & Vicente, J. Universal Behavior of Extreme Price Movements in Stock Markets. *PLoS ONE* **4** (2009).
